# Surgical techniques for Bone Biopsy in Diabetic Foot Infection, and association between results and treatment duration

**DOI:** 10.7150/jbji.45338

**Published:** 2020-06-23

**Authors:** Eric Senneville, Donatienne Joulie, Nicolas Blondiaux, Olivier Robineau

**Affiliations:** 1 Infectious Diseases Department Gustave Dron Hospital F-59200 Tourcoing, and Lille University F-59000, Lille, France.; 2 Northern-West French National Referent Centre for Complex Bone and Joint Infections (CRIOAC Lille-Tourcoing).; 3 Orthopaedic Surgery Department G. Dron Hospital Tourcoing F-59200 Tourcoing France.; 4 Microbiology Laboratory G. Dron Hospital Tourcoing F-59200 Tourcoing France.

**Keywords:** diabetic foot osteomyelitis, bone margins biopsy, outcome, antibiotic therapy, surgery

## Abstract

Surgery is an important part of the management of patients diagnosed with DFO. It consists in some selected patients, to remove all or part of the infected bone(s) or even to amputate all or part of the foot. Despite the use of sophisticated imaging techniques, it is however difficult to remove all the infected tissue while respecting the principles of an economical surgery. Bone biopsy performed at the margins of the resection permits to identify residual osteomyelitis and to adjust the post-surgical antibiotic treatment. Some recent studies have reported the way to perform bone margin biopsies and have assessed the impact of the bone results on the patient's outcome. However, the real impact of a residual osteomyelitis on the risk of recurrent DFO is still debated and questions regarding the interpretation of the results remain to be solved. Similarly, the consequences in terms of choice and duration of the antimicrobial treatment to use in case of positive bone margin are not clearly established.

## Introduction

Osteoarticular infections occur in 20 to 60% of diabetic foot infections and profoundly worsen the outcome of the patients 1. Removal of infected bones has a major place in the management of diabetic foot osteomyelitis (DFO) and has been considered until recently as the unique therapeutic approach 2. Some authors, however, recently reported the results of clinical studies suggesting that a medical approach (i.e., without bone resection) could arrest the infection 3. Experience from different teams showed that this medical approach only applies to some selected patients and that surgery is still proposed to most patients with DFO 1, 2. Surgical management of DFO has the advantage over the medical approach that the removed infected bones do not need any treatment, and this is of importance given the difficulties in obtaining the sterilisation even a stabilisation of the bone infection. Indeed, DFO associates some risk factors for bad outcome such as the frequent peripheral artery disease, impaired of phagocytic functions and the presence of peripheral neuropathy which participates in delaying the wound healing.

The main limitation of the surgical part of the treatment of DFO is the uncertainty about the persistence of residual osteomyelitis following bone resection. Indeed, if all the infected bone tissues have been removed, the infection is no longer an osteomyelitis (or an osteitis) and can therefore be treated as a soft tissue infection (except periarticular structures such as tendons and ligaments). This is of importance since the prognosis of non-bone infections is better and the antibiotic therapy is easier regarding the choice of the antibiotic regimens and their duration than that of DFO 3.

The aim of the present narrative literature review is to provide readers with up-to-date knowledge on the different surgical approaches for DFO and the consequences of bone examination results (i.e., histology and culture) in terms of antibiotic treatment. The summary of the recommendations/current state of knowledge regarding surgical bone biopsy in patients treated for DFO is presented in Table 1.

## Surgical treatment of DFO

Surgical removal of the entire infected bone has been considered in the past and even recently as the standard treatment 2 in patients with DFO because of the poor results obtained by antibiotics alone in these settings. Despite long standing discussions, surgery remains necessary in many (but not all) diabetic foot infections involving bone and joint structures 1,2. One must however keep in mind that the story does not end as soon as the operative wound is closed. Indeed, the surgical resection of the infected bone tissues cannot remove all the bacteria involved in most of the cases. This means that the surgical management of DFO necessarily includes an antibiotic therapy the choice and the duration of which will be determined by the culture results of the intraoperative samples. In other words, surgery and medical parts of the management of DFO are linked by the results of bone specimen examination. This is true even in case of major amputations since several studies have shown that bone biopsies of the margins of the amputation are positive in a non-neglectable proportion of cases 4,5.

Surgery is the unique means to drastically reduce the amounts of bacteria present in bones and sometimes in contiguous tissues (e.g., in case of an abscess). Surgery consists in these settings in draining pus and removing economically all necrotic tissues 6. It can also remove necrotic tissues which antibiotics cannot reach or treat efficiently. Surgery is helpful to remove bacteria in biofilm, which is an important limitation for the antibiotic therapy to be effective. This type of surgery also needs to consider (i) if the coverage of the dehiscence due to resection is feasible, (ii) what is the best level of amputation and (iii) the vascular status of the limb. Given the usual complexity of surgery in these settings, only the most interested and experienced surgeons should be involved 6.

The IWGDF guidance on diabetic foot infection proposes to favour a surgical approach of DFO in case of systemic signs of infection, substantial cortical destruction, osteolysis, macroscopic bone fragmentation (sequestration), an exposed bone within a forefoot ulcer, open or infected joint space and when the patient has prosthetic heart valves 1. Importantly, while surgery may be urgently needed in severe soft-tissue infections, osteomyelitis of the diabetic foot is not a reason for either urgent bone resection or amputation. The usual origin of severe and acute complications of DFIs (i.e., gangrene, septicaemia, and septic shock) is soft-tissue infections rather than osteomyelitis. In order to limit the foot biomechanical consequences of aggressive surgical approaches, some teams have proposed the concept of conservative surgery which consists of a bone resection limited to the infected tissues 7-9. In case of metatarsophalangeal advanced destruction, a resection of the joint is performed without amputation of the whole ray. By keeping the toe in place, it is expected that the adjacent toes are less likely to be deformed due to the dead space created by the missing toe. In addition, this surgery permits to remove the cause of the ulcer at the origin of the soft tissue infection and its spread to the underlying osteo-articular structures. Ha Van *et al*. showed that limited resection (i.e., “conservative surgery”) of the infected phalanx or metatarsal bone under the wound, together with removal of the ulcer site, was effective to obtain complete wound healing 7. Aragon-Sanchez *et al*. reported a consecutive series of 185 diabetic patients with osteomyelitis of the foot and histopathological confirmation of bone involvement, all treated surgically, including 91 conservative surgical procedures 10. Conservative surgery was successful in almost half of the cases and risk factors for failure were exposed bone, the presence of ischemia and necrotizing soft tissue infection 10.

Conservative surgery does not entirely the risk of transfer syndrome as shown by Aragon-Sanchez et al. who reported new episodes of osteomyelitis in 16.9% of the patients 9.

## Bone margin biopsy in patients operated for DFO

Relapsing osteomyelitis episodes observed in patients operated for a DFO are not univocal. The new osteomyelitis may be in relation with a new episode of infection of the initial foot ulcer or at the adjacent rays in relation with the transfer syndrome. Another cause may be the absence of sterilization of the initial infected site although remission of DFO can be obtained despite the complete excision of the infected bone. The rate of remission may however be lower when there is a residual bone infection 11. It seems therefore important to determine whether there is a persistent osteomeylitis following surgery and if additional antibiotic therapy is needed. The use of bone margin biopsy during bone resection or amputation in patients operated for DFO has been reported by some authors. Kowalski et al. studied the rate of residual osteomyelitis in patients who underwent surgery for the treatment of DFO 12. They noted that 35.1% of patients had positive culture of the samples taken at bone margins which correlated with partial metatarsal amputations. They found no differences between patients with negative versus positive histopathologic margins in the primary outcome of definite failure which was defined as an infection relapse at the proximal amputation site. Patients with a positive bone margin were found to have a higher risk of re-amputation 12. Atway et al. reviewed the medical charts of 27 out of 184 diabetic patients who had undergone toe, partial metatarsal, or transmetatarsal amputation 13. To be included in their study, a bone sample had to be taken at the level proximal to the metatarsal amputation or disarticulation at the metatarsophalangeal joint in a standardized fashion, and the amputation had to be primarily closed. The diagnosis of DFO was based on standard imaging modalities, including plain X-rays, magnetic resonance imaging, and/or nuclear bone scan. Based on bone margins culture, DFO was diagnosed in 11 patients (40.7%) including 23.1% (3/13) in patients who underwent toe amputation and 57.1% (8/14) in patients who underwent partial metatarsal or trans-metatarsal amputation (p=0.12). Almost half of the patients had a poor outcome (13/27, 48.2%). Failure was more frequent in patients with positive versus negative culture of the bone margin biopsy (81.8% (9/11) versus 25% (4/16), respectively p=0.0063) 15. Fuji et al. reported a series of 28 patients with DFO who were operated with examination of the proximal margins of resected bones with histopathology to ascertain whether osteomyelitis was completely resected 14. Complete healing rate of the foot wound was 100% in the non-ischemic patients with negative bone margin cultures versus 84.6% in the ischemic patients 14. Patients with positive bone margins had significantly more elevated CRP values in the pre and post-operative periods and a higher chance to be re-operated (100% versus 15.1%; p<0.001). In another large retrospective study of 72 patients operated for DFO, Schmidt et al. compared the characteristics of patients with or without “dirty surgical margin“(i.e., residual osteomyelitis with positive culture and/or histology) 15. They found that lower preoperative albumin, positive probe-to-bone test, large surface of the foot ulcer, lower toe-pressure, and higher red cell mean volume were all associated with a residual osteomyelitis on bone margins. They also found that the obtainment of clean margins (i.e. no residual osteomyelitis) was associated with better outcomes including lower rates of wound dehiscence, reulceration, and amputation. In addition, they also found that patients with residual osteomyelitis were 2.6 times more likely to be readmitted (p=0.08).

In a series of 66 cases of amputations defined as surgical removal of bone for DFO, 39 (59%) had remission at 12-month follow-up 16. Remission was reported more frequently in patients amputated at the digit level as compared to metatarsal level (p=0.045). Remission and failure cases did not differ regarding the presence or absence of histopathologic features of osteomyelitis at the bone margins (p=0.72) which is consistent the results of two other recent studies 17, 18.

## Bone margin biopsy in practical

The dorsal and plantar approaches to metatarsal head resections in patients with DFO have been compared regarding the recovery time and the development of complications 19. The authors reviewed the medical chart of 108 patients including 53 with a plantar approach and 55 a dorsal approach. While healing time was similar in the two groups, the patients undergoing a dorsal approach developed more post-surgical complications than patients treated through a plantar approach. In the case of exarticulation, Mijuskovic et al. propose to take two bone cylinders (1 for culture and 1 for histological analysis) from the metatarsal head using an 8G Jamshidi biopsy needle 20. In the case of transmetatarsal amputation, they took a 3 to 5-mm slice of cortico-cancellous metatarsal bone by the means of an oscillating saw. The slice was then cut in half, with 1 half used for culture and the other used for histology. In an attempt to reduce the rate of positive proximal margins and to resect all infected bone using pre-operative MRI measurements, Bernstein et al. proposed to perform a 1 cm resection margin proximal to the metatarsal osteomyelitis 21. By using this technique, the authors obtained a positive proximal margin rate of 9% which is a lower proportion when compared to the 30 to 40% reported in the literature. It must be noted that the consequences of larger resection on the foot biomechanics need to be evaluated and that this procedure needs to perform a preoperative MRI in every patient.

Two different types of bone resection should be considered, one is the amputation of a ray that can be at a metatarsal or phalangeal level creating one bone stump and the other is a resection of a joint (i.e., the conservative surgery) in which there are two bone stumps. During the amputation, a bone margin biopsy is recommended while it is less clear that a biopsy should be done at each bone stump in case of conservative surgery. A bone biopsy performed at each stump appears to be ideal as there is no apparent reason which supports that the infection would only spread to proximal or distal direction. On the other hand, a bone biopsy at both stumps is likely to complicate the intervention. In case of a trans-articular amputation, it seems more appropriate to remove the cartilage which is avascular and therefore less able to defend itself against the pathogens. Whatever the type of surgery, it is important to take a bone sample with proper precautions to avoid the contamination of the samples i.e., by changing gloves, using sterile surgical instruments and a no-touch technique during the biopsy. External needle biopsy does not seem adequate since the level of amputation is not known with precision before the intervention and is not suitable for obtaining a bone margin biopsy.

## Bone sample examinations

While it is recommended to use both histology and culture to affirm the diagnosis of osteomyelitis 1 one must admit that this is rarely done in the daily practice and the diagnosis is often only based on one of the two 3. For clinical research on DFO, it seems important to define a bone involvement by using both histology and culture and to require both positive examinations. In the daily practice, the interest of histology is questionable as it may take a long time before the results to become available. In addition, there is no consensual definition of DFO regarding histology features. Possible/definitive osteomyelitis was defined by Mijuskovic et al. as the visualisation at any high-power field of at least one/five neutrophilic granulocyte, in combination with marrow necrosis, fibrosis, lympho-plasmocytic infiltration, oedema, or reactive bone formation 20. Osteomyelitis was excluded if the analysed fields did not contain any neutrophilic granulocytes. Inflammation or necrosis of bone, or leukocyte infiltrate at the margin of resection are the most frequent abnormalities reported by the authors to be indicative of an osteomyelitis 20,21. The results of the matched case-control study reported by Weiner et al. provide data in favour of using culture rather than histology 22. The authors compared histology and culture to make the diagnosis of DFO on 44 bone specimens taken surgically in 37 patients. The proportion of bone samples positive for culture and histology was 70.4% and 72.3%, respectively. Bone biopsies positive for histology were also positive for culture in 75% of the cases and positive culture but negative histology was seen in only 7 cases (16%). Microbiology achieved a sensitivity/specificity/positive predictive value/negative predictive value of 0.75/0.42/0.77/0.39, respectively and a diagnostic accuracy of 0.66. It was concluded by the authors that culture performed as well as did histology for diagnosing DFO 22. The major difference is that culture provides the physician with useful data regarding the antibiotic treatment. When there is paucity of material to be sampled, it seems therefore logical to renounce on histology and stick with microbiology.

We are unaware of any studies that assessed the concordance of pathogens between the amputated bone and the residual stump microbiology. Another point in the daily practice is the difficulty to interpret the culture results especially when bacteria from the skin flora have been identified which may indicate a possible contamination of the bone samples. This issue has been addressed in a recent paper from Mijuskovic et al. who reported a series of 51 consecutive patients operated for toe or forefoot amputation 20. Histological examination and culture were both performed on bone samples taken at the residual bone margins. Positive culture and histology were recorded in 33 (65%) and 14 (27%) patients, respectively. Discordant results (i.e., positive culture but negative histology) was found in 21 (41%) of the patients which led the authors to state that bone culture in this setting has a high false-positive rate provided histology is considered the reference to rule out osteomyelitis. The authors respected appropriate measures to reduce the risk of contamination of the bone samples which were taken at the end of the intervention using a new set of surgical instruments. The reasons for such a high proportion of apparently false positive culture are not clear but similar results were reported by another group 12. The high rate of positive cultures in association with negative histological findings in biopsy specimens was interpreted by the authors as being due to intraoperative bacterial contamination of the specimens. In other hand, Crisologo and colleagues determined that 16S rRNA gene testing identified more cases of DFO compared to histology and traditional culturing 23. The presence of slow-growing bacteria in biofilms may be the reason for culture-negative findings 24. In a prospective study Malone et al. compared the results of conventional culture versus next-generation DNA sequencing for 14 consecutive bone specimens (i.e., bone margins and infected bone) taken during surgery for DFO 25. Eight of 14 (57%) proximal bone margins had no growth by conventional culture but were identified in all proximal bone specimens by DNA sequencing. In addition, using a combination of scanning electron microscopy and peptide nucleic acid fluorescent in situ hybridization, they identified that bacteria can still reside in what seems to be proximal 'clean' margins. This study did not however include histology examination of the bone specimens.

White et al. prospectively studied a series of 25 patients with a suspicion of DFO who had combined histologic and microbiologic evaluation of percutaneous bone biopsies 26. Sixteen biopsies were positive for histology and eight of them were culture positive, seven of the eight culture-negative were positive for histology. No patients were recorded for positive culture and negative histology. The sensitivity of culture for the diagnosis of DFO was 42% and the sensitivity of the combination of culture and histology was 84%. The authors concluded that histology examination should be performed on biopsies taken percutaneously for a suspicion of DFO.

In their recent study, Schmidt et al. sent the bone margin specimens to two board-certified pathologists with expertise in bone pathology who both used a strict classification of histopathology definitions for the diagnosis of osteomyelitis 18. By standardizing the definitions and interpretation of histopathology for the distal surgical margin, the authors reported a high kappa coefficient of 0.96 which however decreased to 0.71 for the evaluation of the proximal surgical margin. The interpretation of the presence of fibrosis seemed to be the cause of the disagreements between the two observers.

## Management of patients with a positive bone margin biopsy

Data on the optimal antibiotic therapy to administer after bone resection of foot amputation in these patients are lacking. Current guidelines recommend treating with antibiotics for up to 6 weeks if the culture demonstrates pathogen(s) or if the histology demonstrates osteomyelitis 1.

The optimal duration of DFO is a difficult subject as only one randomized controlled study has addressed this question 27. The results of this study suggested that not more than 6 weeks were necessary for the medical treatment of DFO. It must be noted however that these patients had neuropathic feet, had had all a transcutaneous bone biopsy and were mostly treated with rifampicin or fluoroquinolones combinations. It is unlikely that the duration of the antibiotic treatment required for an osteomyelitis of the foot that has been operated with partial resection (i.e., conservative surgery) or even amputation should be as long as it is for patients treated non-surgically. In the study from Lazaro-Martinez et al. it was suggested that 10 days following surgery was enough. Bone margins were however, not examined in the patients treated surgically 28. Less than 7 days of directed antibiotic therapy for a positive bone margin culture was associated with a higher risk of failure in the study from Barsches et al.

In the study from Johnson et al. the mean ± SD antibiotic duration was 37.6 days ± 24.1 versus 17.7 days ± 29.6 (p=0.001) in patients with positive and negative bone margins, respectively 15. Mijuskovic et al. treated their patients with residual osteomyelitis with postoperative intravenous antibiotic for 2 weeks followed by oral treatment, usually for an additional 10 weeks 20.

The presence of bacterial biofilms may be of importance regarding the choice of the antimicrobial treatment given the differences in efficacy of antibiotics against planktonic and slow-growth bacteria (e.g. rifampicin versus beta-lactams which only work against multiplying bacteria) 29-31.

As many of the bone biopsies contain bacteria embedded into a biofilm, this may explain why rifampicin and fluoroquinolone combination regimens (i.e., those with the highest antibacterial activity into the biofilm environment) are likely to be associated with a better outcome of the patients 31, 32. However, these antibiotic regimens are not always well tolerated due to gastro-intestinal problems or drug-drug interactions and it is therefore important to limit their prescription to biofilm infections (i.e., DFO without bone resection). The interest of antibiofilm antibiotic regimens for bone margin specimens containing biofilm infection has not been studied yet.

## Summary

Bone resection and minor even major amputations are unlikely to remove all the bacteria involved and it seems therefore important to obtain data on the persistence of infected tissues including bone. Most but not all the studies have reported a negative effect of residual osteomyelitis on the outcome of patients operated for DFO. A lot of questions about the examination of the bone specimens (e.g., culture or/and histology, the interpretation of the results) have not yet been solved. Some studies have pointed out the discrepancies between culture and histology of bone margin biopsies. These studies suggest that some of the positive cultures of bone margin biopsies are in fact in relation with contaminated specimens. Histology may help interpret the results of bone margin biopsies but the delay for obtaining the results in the daily practice appears to be a serious limitation in its routine implementation. In addition, the reliability of histology in these settings is an issue as suggested in some studies. The type and duration of the antibiotic treatment to consider in patients with a persistent osteomyelitis on the bone margins are currently unknown.

In conservative surgery, bone specimens should probably be taken at both proximal and distal margins which, however, complicates the procedure, especially if both histology and culture are performed systematically. The role of molecular genetic techniques in the detection of bacteria in bone margin biopsies is not clearly established. These techniques do not provide information on the antimicrobial susceptibility profile of the bacterial strains identified. In the absence of indication regarding the pathogenic role of the organisms identified by these techniques, no recommendations have been made so far on their use in these settings. Data on the choice and duration of the antibiotic treatment to prescribe following bone margin biopsy are lacking. The first question is about the use of empirical post-biopsy antibiotic therapy while waiting for the results. Given that a bone and joint resection or even an amputation of all or part of the foot has just been performed in a context of a possible infection, it seems logical to consider an empirical antibiotic therapy started intraoperatively after the tissue samples have been taken and continued until the results of culture are available. As some of these patients are operated while receiving antibiotics a negative result should be interpreted with caution. If a bacterial documentation has been obtained prior to the surgery and histology is positive antibiotics should probably be administered and should be stopped if histology is negative. This raises the potential interest of histology examination in these settings. In the cases of negative culture and positive histology without previous documentation, antibiotics should be chosen taking account of the antibiotics which led to a negative culture. The duration is another difficult as the optimal duration of antibiotic treatment for DFO is not known. The last edition of the IWGDF recommends consider treating DFO with antibiotic therapy for no longer than 1-2 weeks if all infected bone has been surgically removed 33. No recommendations are however provided in cases of positive bone margin biopsy.

Additional clinical studies are necessary to answer some unsolved questions about surgical bone biopsy in the setting of DFO. Some standards for further clinical studies should be reported such as the description and gradation of soft tissue infection, the morphology and location of the tissue defect, details on concomitant peripheral artery disease, on severity of diabetic neuropathy, on surgical technique of amputation and wound closure, and on pre- and postoperative antibiotic treatment. These studies should address (i) which clinical intraoperative findings warrant the retrieval of a bone biopsy, (ii) how should a bone biopsy be retrieved (e.g., the minimum size and the optimal number of the bone samples), (iii) which histology criteria for the presence of acute or chronic osteomyelitis should be used and (iv) how should the biopsy be handled for microbiology assessment.

## Figures and Tables

**Table 1:** Recommendation/current state of knowledge regarding surgical bone biopsy in patients with diabetic foot osteomyelitis (DFO)

Questions	Recommendations/current state of knowledge
Should bone biopsy be performed in every patient with a (suspected) DFO?	It is useful to obtain a bone specimen in almost all cases of (suspected) DFO However, bone biopsy is not always feasible due to lack of time and experience Bone biopsy seems most important to perform in case of difficulties in identifying the causative pathogen(s) or its (their) antibiotic susceptibility Bone biopsy may not be needed if a deep tissue specimen grows a single virulent pathogen, especially *S. aureus*.
Should culture and histology be systematically performed on bone samples?	The diagnosis of DFO is established when one or more bone specimens has *both* a positive culture and characteristic histopathological findings. Culture provides useful data for guiding the choice of the antibiotic treatment Histology is useful in patients on antibiotic therapy because of the risk of false negative culture In case of limited amount of bone material, it is better to only consider culture than histology as both seem to perform equally in terms of diagnosis accuracy.
Should bone biopsy be performed on each stump in case of a conservative surgery (i.e., joint resection)?	Since the infectious process is likely to have spread towards both distal and proximal direction from the resected bone, performing a biopsy on both stumps seems appropriate.
In case of exarticulation, should the cartilage be removed?	The avascular cartilage material is less able to defend itself against the pathogens (especially *S. aureus*) and should be removed in case of exarticulation.
What is the optimal duration of the antibiotic therapy in case of positive bone margin culture in case of conservative surgery or amputation?	3-week duration seems enough in these situations 1 to 2-week duration should be enough for patients in whom all infected bone has been resected.
Are rifampicin combinations recommended in patients with a bone margin biopsy positive in culture for *Staphylococcus* sp.?	In case of conservative surgery or amputation, the presence of biofilm in residual bone tissues is unlikely and therefore is not in favour of the use of rifampicin combinations Other antibiotics with high bone diffusion can be used such as clindamycin, tetracyclines, fluoroquinolones (in combination), cotrimoxazole, linezolid, fusidic acid (in combination)
What are the histology criteria for the presence of acute or chronic osteomyelitis?	Acute osteomyelitis is defined by necrosis, destroyed bone and infiltration of polymorphonuclear granulocytes usually associated with congestion or thrombosis of medullary or periosteal small vessels. Chronic osteomyelitis is characterized by destroyed bone and infiltration of lymphocytes, histiocytes and/or plasmatic cells In all cases of osteomyelitis, areas of fibrosis are described in variable forms as well as medullar edema.

## References

[1] Lipsky BA, Berendt AR, Cornia PB, et al; Infectious Diseases Society of America (2012). 2012 Infectious Diseases Society of America clinical practice guideline for the diagnosis and treatment of diabetic foot infections. Clin Infect Dis.

[2] Pinzur MS (2019). What are the Optimal Cutoff Values for ESR and CRP to Diagnose Osteomyelitis in Patients with Diabetes-related Foot Infections?. Clin Orthop Relat Res.

[3] Senneville E, Robineau O (2017). Treatment options for diabetic foot osteomyelitis. Expert Opin Pharmacother.

[4] Vaznaisiene D, Beltrand E, Laiskonis AP (2013). Major amputation of lower extremity: prognostic value of positive bone biopsy cultures. Orthop Traumatol Surg Res.

[5] Vaznaisiene D, Sulcaite R, VitkauskieneA (2015). Section's osseous slice biopsy during major amputation of lower extremity: preliminary results of prospective cohort study. BMC Inf Dis.

[6] Aragon-Sanchez J (2010). Treatment of diabetic foot osteomyelitis: a surgical critique. Int J Low Extrem Wounds.

[7] Ha Van G, Siney H, Danan JP (1996). Treatment of osteomyelitis in the diabetic foot. Contribution of conservative surgery. Diabetes Care.

[8] Molines-Barroso RJ, Lázaro-Martínez JL, Aragón-Sánchez J (2013). Analysis of transfer lesions in patients who underwent surgery for diabetic foot ulcers located on the plantar aspect of the metatarsal heads. Diabet Med.

[9] Aragon-Sanchez J, Lazaro-Martinez JL, Hernandez-Herrero C (2012). Does osteomyelitis in the feet of patients with diabetes really recur after surgical treatment? Natural history of a surgical series. Diabet Med.

[10] Aragón-Sánchez FJ, Cabrera-Galván JJ, Quintana-Marrero Y (2008). Outcomes of surgical treatment of diabetic foot osteomyelitis: a series of 185 patients with histopathological confirmation of bone involvement. Diabetologia.

[11] Hachmoller A (2007). Outcome of minor amputations at the diabetic foot in relation to bone histopathology: a clinical audit. Zentralbl Chir.

[12] Kowalski TJ, Matsuda M, Sorenson MD (2012). The effect of residual osteomyelitis at the resection margin in patients with surgically treated diabetic foot infection. J Foot Ankle Surg.

[13] Atway S, Nerone VS, Springer KD (2012). Rate of residual osteomyelitis after partial foot amputation in diabetic patients: a standardized method for evaluating bone margins with intraoperative culture. J Foot Ankle Surg.

[14] Fujii M, Terashi H, Yokono K (2016). Surgical treatment strategy for diabetic forefoot osteomyelitis. Wound Repair Regen.

[15] Schmidt BM, McHugh JB, Patel RM (2019). Prospective Analysis of Surgical Bone Margins After Partial Foot Amputation in Diabetic Patients Admitted With Moderate to Severe Foot Infections. Foot Ankle Spec.

[16] Johnson MJ, Shumway N, Bivins M (2019). Outcomes of Limb-Sparing Surgery for Osteomyelitis in the Diabetic Foot: Importance of the Histopathologic Margin. Open Forum Infect Dis.

[17] Beieler AM, Jenkins TC, Price CS (2012). Successful limb-sparing treatment strategy for diabetic foot osteomyelitis. J Am Podiatr Med Assoc.

[18] Barshes NR, Mindru C, Ashong C (2016). Treatment failure and leg amputation among patients with foot osteomyelitis. Int J Low Extrem Wounds.

[19] Tardaguila-Garcia A, Sanz-Corbalan I, Molines-Barroso RJ (2019). Complications associated with the approach to metatarsal head resection in diabetic foot osteomyelitis. Int Wound J.

[20] Mijuskovic B, Kuehl R, Widmer AF (2018). Culture of bone biopsy specimens overestimates rate of residual osteomyelitis after toe or forefoot amputation. J Bone Joint Surg Am.

[21] Bernstein B, Stouder M, Bronfenbrenner E (2017). Correlating pre-operative MRI measurements of metatarsal osteomyelitis with surgical clean margins reveals the need for a one centimeter resection margin. J Foot Ankle Res.

[22] Weiner RD, Viselli SJ, Fulkert KA (2011). Histology versus microbiology for accuracy in identification of osteomyelitis in the diabetic foot. J Foot Ankle Surg.

[23] Crisologo PA, La Fontaine J, Davis K (2018). Are we misdiagnosing diabetic foot osteomyelitis?. Diab Care.

[24] Costerton JW, Post JC, Ehrlich GD (2011). New methods for the detection of orthopedic and other biofilm infections. FEMS Immunol Med Microbiol.

[25] Malone M, Fritz BG, Vickery K (2019). Analysis of proximal bone margins in diabetic foot osteomyelitis by conventional culture, DNA sequencing and microscopy. APMIS.

[26] White LM, Schweitzer ME, Deely DM (1995). Study of osteomyelitis: utility of combined histologic and microbiologic evaluation of percutaneous biopsy samples. Radiology.

[27] Tone A, Nguyen S, Devemy F (2015). Six-week versus twelve-week antibiotic therapy for nonsurgically treated diabetic foot osteomyelitis: a multicenter open-label controlled randomized study. Diab Care.

[28] Lázaro-Martínez JL, Aragón-Sánchez J, García-Morales E (2014). Antibiotics versus conservative surgery for treating diabetic foot osteomyelitis: a randomized comparative trial. Diabetes Care.

[29] Johani K, Fritz BG, Bjarnsholt T (2019). Understanding the microbiome of diabetic foot osteomyelitis: insights from molecular and microscopic approaches. Clin Microbiol Infect.

[30] Lavigne JP, Sotto A (2017). Microbial management of diabetic foot osteomyelitis. Future Microbiol.

[31] Senneville E, Lombart A, Beltrand E (2008). Outcome of diabetic foot osteomyelitis treated non-surgically. Diab Care.

[32] Wilson BM, Bessesen MT, Doros G (2019). Adjunctive Rifampicin therapy for diabetic foot osteomyelitis in the veterans health administration. JAMA Netw Open.

[33] Lipsky BA, Senneville E, Abbas ZG International Working Group on the Diabetic Foot (IWGDF) Infection Guideline 2019. www.iwgdfguidelines.org.

